# A Mutation in *CACNA1S* Is Associated with Multiple Supernumerary Cusps and Root Maldevelopment

**DOI:** 10.3390/diagnostics13050895

**Published:** 2023-02-27

**Authors:** Piranit Kantaputra, Niramol Leelaadisorn, Athiwat Hatsadaloi, Natalina Quarto, Worrachet Intachai, Sissades Tongsima, Katsushige Kawasaki, Atsushi Ohazama, Chumpol Ngamphiw, Paswach Wiriyakijja

**Affiliations:** 1 Center of Excellence in Medical Genetics Research, Faculty of Dentistry, Chiang Mai University, Chiang Mai 50200, Thailand; 2 Division of Pediatric Dentistry, Department of Orthodontics and Pediatric Dentistry, Faculty of Dentistry, Chiang Mai University, Chiang Mai 50200, Thailand; 3 Dental Department, Rot-et Hospital, Roi-et 45000, Thailand; 4 Dental Home Clinic, Khon Kaen 40000, Thailand; 5 Division of Plastic and Reconstructive Surgery, Department of Surgery, School of Medicine, Stanford University, Stanford, CA 94305, USA; 6 National Biobank of Thailand, National Science and Technology Development Agency (NSTDA), Thailand Science Park, Pathum Thani 12120, Thailand; 7 Division of Oral Anatomy, Faculty of Dentistry & Graduate School of Medical and Dental Sciences, Niigata University, Niigata 951-8514, Japan; 8 Department of Oral Medicine, Faculty of Dentistry, Chulalongkorn University, Bangkok 10330, Thailand; 9 Avatar Biotechnologies for Oral Health and Healthy Longevity Research Unit, Chulalongkorn University, Bangkok 10330, Thailand

**Keywords:** calcium homeostasis, calcium influx, enamel knot, root maldevelopment, supernumerary cusps, single-rooted molars, taurodontism, terotogenic effect

## Abstract

Background: Enamel knots and Hertwig epithelial root sheath (HERS) regulate the growth and folding of the dental epithelium, which subsequently determines the final form of tooth crown and roots. We would like to investigate the genetic etiology of seven patients affected with unique clinical manifestations, including multiple supernumerary cusps, single prominent premolars, and single-rooted molars. Methods: Oral and radiographic examination and whole-exome or Sanger sequencing were performed in seven patients. Immunohistochemical study during early tooth development in mice was performed. Results: A heterozygous variant (c. 865A>G; p.Ile289Val) in *CACNA1S* was identified in all the patients, but not in an unaffected family member and control. Immunohistochemical study showed high expression of Cacna1s in the secondary enamel knot. Conclusions: This *CACNA1S* variant seemed to cause impaired dental epithelial folding; too much folding in the molars and less folding in the premolars; and delayed folding (invagination) of HERS, which resulted in single-rooted molars or taurodontism. Our observation suggests that the mutation in *CACNA1S* might disrupt calcium influx, resulting in impaired dental epithelium folding, and subsequent abnormal crown and root morphology.

## 1. Introduction

Development of a tooth requires several signaling centers and a series of developmental events. Variation in signaling pathways might have effects on tooth number and morphology [1]. Tooth formation initiates around the 6th or 7th week of human gestation as continuous thickening of the oral ectoderm, called dental lamina or odontogenic bands, in the upper and lower jaws. This dental lamina indicates where the future teeth will form [2,3]. Subsequently, dental lamina, which expresses a number of signaling centers involving transcription factors and signaling pathways, such as SHH, FGF, and BMP, regulates the invagination process of the dental epithelium [1]. The invagination of the dental epithelium into the underlying neural crest-derived mesenchyme generates tooth placodes at specific positions, which mark the onset of tooth morphogenesis. Tooth germs are formed by the activity of sequential epithelial-mesenchymal interactions via numerous signaling pathways and go through a series of developmental stages prior to mineralization. Epithelial cells within the tooth placodes proliferate and form the bud-shaped structure surrounded by the condensed dental mesenchyme [2,3]. Initially the intrinsic potential to form a tooth is the dental epithelium and subsequently switches to the underlying mesenchyme during later stages of development [2]. The determination of tooth region, tooth type, crown morphology, and individual cusps is regulated by a large number of signaling molecules that have influence on differential tissue growth and differentiation [4].

Each tooth is highly self-regulated. The final crown morphology is the product of pre-determined epithelial morphogenesis during the cap and bell stages and involves rapid cell proliferation precisely regulated in time and space, as well as the folding of the dental epithelium at the sites of the future tooth cusps [4]. The primary and secondary enamel knots, which comprise non-dividing cells, are thought to direct the differential growth and subsequent folding of the inner dental epithelium. A number of FGF molecules are implicated in the control of cell proliferation around the non-dividing primary and secondary enamel knots [4]. Ectodysplasia (*Eda*) has a major influence on the spatial formation of the successional signaling centers during the process of tooth formation. It controls the size of enamel knots, while a lack of *Eda* results in a reduction in enamel knots, abnormal enamel organ, and, subsequently, the abnormal tooth is formed. This phenotype can be rescued by the FGF10 molecule [5,6]. Increased expression of *Eda* (increased ectodysplasin signaling) in mice results in an increased number of cusps, changes in shape and position of cusps, and subsequent increased number of teeth [7]. The number and spatial patterning of tooth cusps are precisely regulated by the iterative activation of secondary enamel knots, which are epithelial signaling centers providing developmental information [8]. The spatiotemporal induction of the secondary enamel knots, which are the key players in tooth cusp patterning and occlusal development, involves repeated activation and inhibition of signaling [4]. The effects of FGF signaling on the inner dental epithelium and the areas of non-dividing cells of secondary enamel knots play roles in folding of the inner dental epithelium, leading to tooth cusp formation and subsequent occlusal development [4,9]. The formation of tooth cusps within a single tooth can be explained by a patterning cascade model, the product of activating and inhibiting activity in sculpturing occlusal morphology [8,10,11,12]. The dental epithelium determines the cusp size and shape, and the dental mesenchyme determines the tooth size. Therefore, the final number of tooth cusps depends on the sizes of the tooth and the previously formed cusps [13]. The morphology and size of the later developing cusps are dependent on the position and size of the earlier forming ones [14,15].

In general, an increase in morphological complexity is apparent throughout evolution [16,17]. However, teeth in patients with genetic mutations or mutant or transgenic mice generally have simpler tooth morphology or less dental complexity [17,18]. Interestingly, a unique set of dental anomalies, including molars with multiple supernumerary cusps, single-cusped premolars, taurodontism, single-rooted molars, and tooth agenesis, has been reported in patients with a heterozygous missense variant (c. 865A>G; p.Ile289Val) in *Calcium Channel voltage-dependent, L-type, Alpha-1S subunit* (*CACNA1S*; MIM 114208) [19]. It is interesting to note that every reported patient with that variant had molars with multiple supernumerary cusps, single-rooted molars, or taurodontism. CACNA1S encodes the pore-forming subunit of the dihydropyridine (DHP) channel. These findings suggest the mutation in *CACNA1S* led to an impairment in calcium influx, abnormal calcium homeostasis, and resulted in teeth with abnormal cusp and root formation. Thus, crown morphology may reflect the morphology of roots.

Here, we report seven new patients from three unrelated Thai families affected with multiple supernumerary cusps, single-cusped premolars, single-rooted molars, taurodontism, and tooth agenesis. Whole-exome and Sanger sequencing identified a heterozygous variant in *CACNA1S*. Molecular findings and gene expression study suggest that the basic pathogenetic mechanism of molars with multiple supernumerary cusps and single roots involves disruptive folding of the dental epithelium as a result of abnormal calcium homeostasis.

## 2. Case Presentation

We recruited patients for genetic study. The inclusion criteria were patients with multiple supernumerary cusps, round-shaped occlusal surface molars, molars with multiple supernumerary cusps, premolars with single prominent cusp, single-rooted or taurodontic molars, and tooth agenesis. Exclusion criteria were patients with normal teeth with no multiple supernumerary cusps or single-rooted molars.

### 2.1. Patients

**Figure 1:**
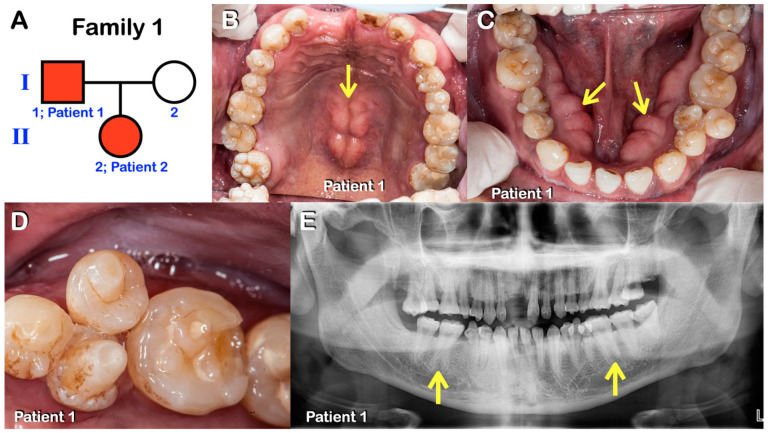
(**A**–**E**) Family 1. (**A**) Pedigree of family 1. Patients 1 and 2. (**B**–**D**) **Patient 1.** Permanent dentition. Round-shaped permanent molars, molars with multiple supernumerary cusps, premolars with single prominent cusps, torus palatinus (arrow in B), and torus mandibularis (arrows in C). Close-up view of mandibular premolars with single prominent cusps. (**E**) Panoramic radiograph showing agenesis of the right permanent mandibular third molar, single-rooted permanent molars, and taurodontism (arrows).

**Figure 2:**
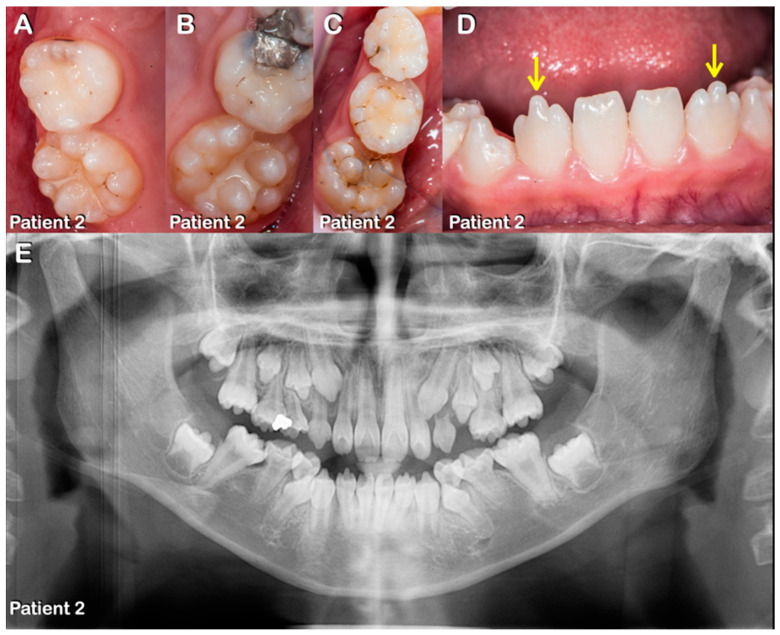
**Patient 2.** (**A**–**E**) Mixed dentition. (**A**–**C**) Round-shaped primary and permanent molars with multiple supernumerary cusps. (**D**) Prominent medial mamelon of the mandibular lateral permanent incisors (arrows). (**E**) Panoramic radiograph showing agenesis of all third permanent molars, single-rooted primary and permanent molars, and taurodontism.

**Figure 3:**
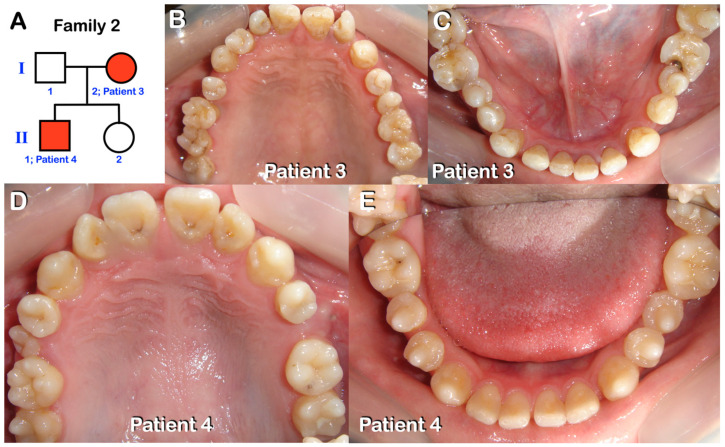
Family 2. (**A**) Pedigree of family 2. Patients 3 and 4. (**B**,**C**) **Patient 3** and (**D**,**E**) **Patient 4.** Both are in permanent dentition. Round-shaped permanent molars, molars with multiple supernumerary cusps, and premolars with single prominent cusps.

**Figure 4:**
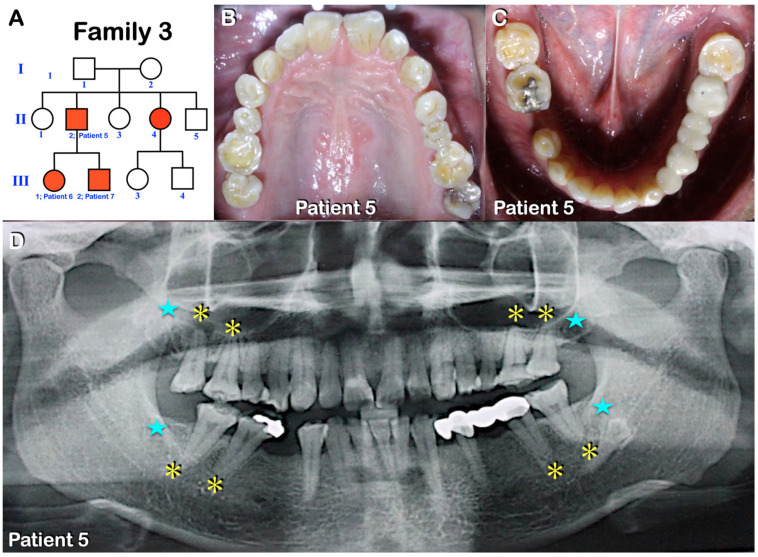
Family 3. (**A**) Pedigree of family 3. Patients 5–7. (**B**,**C**) Patient 5. Permanent dentition. Round-shaped permanent molars, molars with multiple supernumerary cusps, and premolars with single prominent cusps. (**D**) Patient 5. Panoramic radiograph showing agenesis of all third permanent molars (blue stars), single-rooted permanent molars, and taurodontism (yellow asterisks).

**Figure 5:**
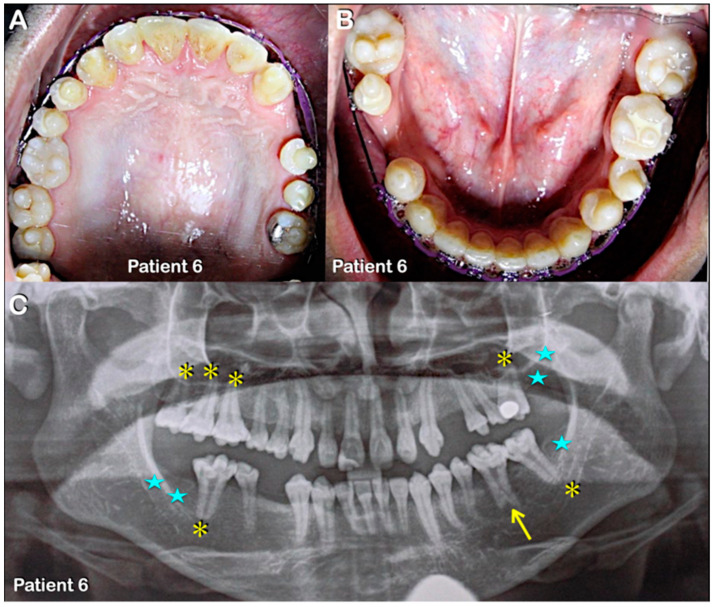
Patient 6. (**A**–**C**) Patient 6. (**A**,**B**) Permanent dentition. Round-shaped permanent molars, molars with multiple supernumerary cusps, and premolars with single prominent cusps. (**C**) Panoramic radiograph showing agenesis of second and third permanent molars (blue stars) and single-rooted permanent molars (yellow asterisks). Note severe taurodontism (yellow arrow).

**Figure 6:**
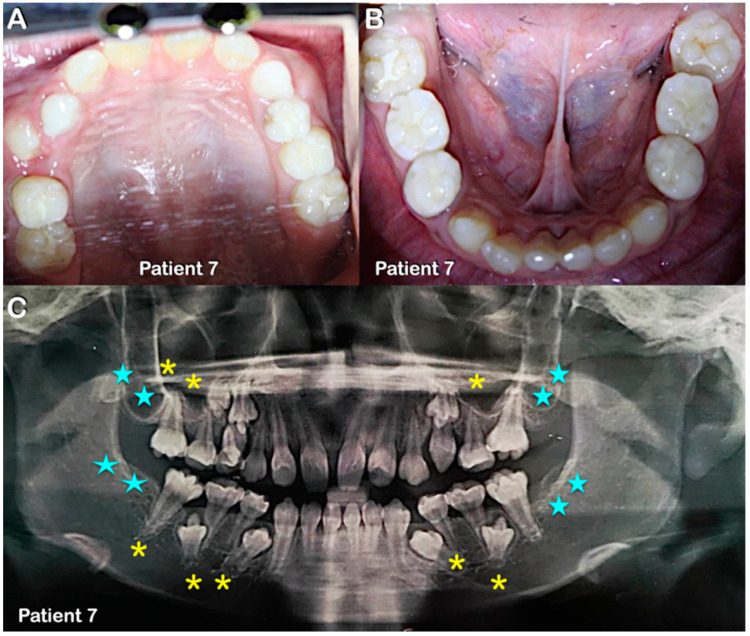
Patient 7 in mixed dentition. (**A**,**B**) Round-shaped permanent molars and molars with multiple supernumerary cusps. (**C**) Panoramic radiograph showing agenesis of second and third permanent molars (blue stars) and single-rooted primary and permanent molars (yellow asterisks).

Patients 1–7 were from three unrelated Thai families (Figure 1, Figure 2, Figure 3, Figure 4, Figure 5 and Figure 6). Patients 2 and 7 had mixed dentition; the remaining five patients were in permanent dentition. All patients share similar dental phenotypes, including round-shaped occlusal surface molars, molars with multiple supernumerary cusps, premolars with single prominent cusp, single-rooted or taurodontic molars, and tooth agenesis. Prominent middle mamelon of the mandibular lateral permanent incisors was observed in patient 2 (Figure 2D).

### 2.2. Whole-Exome and Sanger Sequencing and Bioinformatic Analysis

Genomic DNA was isolated from saliva using Oragene-DNA (OG-500) Kit (DNA Genotek, CANADA). Using the targeted capture kit, SureSelect V6 (PR7000-0152; Agilent Technologies, CA, USA), whole-exome sequencing (WES) of an affected father (patient 1) and his affected daughter (patient 2) from family 1 was carried out Macrogen Inc. (Seoul, Korea) provided the WES service with 80% coverage of the exonic target regions guaranteed at 30× depth. The germline variant discoveries were predicted by using Genomics analysis toolkit (GATK) version 3.8.1. The alignment of the raw sequencing data, FASTQ files, to the reference sequence GRCh37v1.6 was carried out using BWA-mem. The functional annotations of these variants were identified by the Variant Effect Predictor (VEP) tool build 102 with the additional plugin and the database of nonsynonymous functional prediction (dbNSFP) version 4.1a. Then, pathogenic variants were prioritized according to inheritance model, functional annotation. Rare variants of interest were identified by standard variant filtering pipelines (allele frequencies < 0.0001), the algorithm Combined Annotation Dependent Depletion (CADD) with scores > 15. Furthermore, variant allele frequencies were determined by comparing against public databases, including gnomAD, 1000G, GenomeAsia, and the Thai Reference Exome database (T-Rex).

Once the variant in *CACNA1S* (NM_000069.2; NP_000060.2) was identified from patients 1 and 2, Sanger sequencing for this variant was performed in patients 3–7 and the unaffected family members (if available), in order to identify if the affected patients had the same variant. The forward and reverse primers for Sanger sequencing (sense: 5′-GGGGATTTCCCCATAGGATGC-3′ and antisense: 5′-TACACCTTTCCTCCTGTCGT-3′) for exon 6 of *CACNA1S* gene were used to amplify the target. The sequence analysis software, Sequencher 4.8 (Genecodes, Ann Arbor, MI, USA) was used to identify the presence of variants and co-segregation between genotype and phenotype within the families.

#### Molecular Findings of Patients

**Figure 7:**
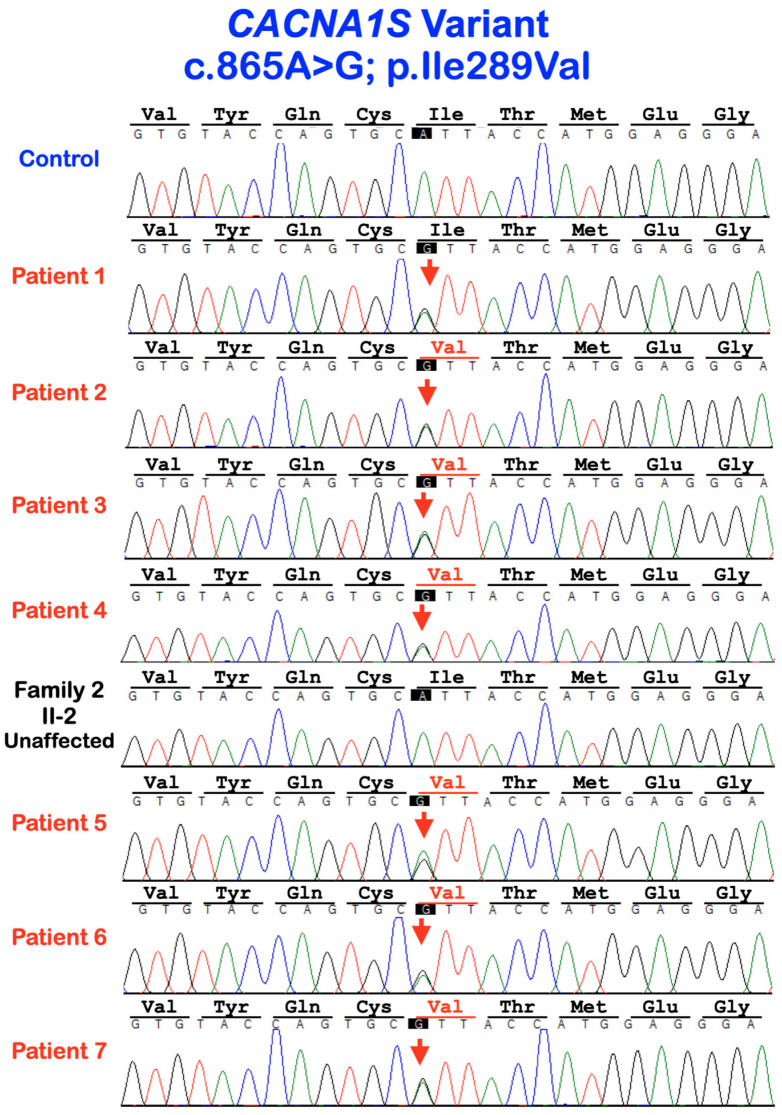
Sequence chromatograms of patients 1–7, the unaffected II-2 of family 2, and a control. The single base substitution from A to G at nucleotide 865 in *CACNA1S* gene is predicted to cause a change in amino acid isoleucine (Ile) to valine (Val) at amino acid residue 289 (c.865A>G; p.Ile289Val).

Whole-exome or Sanger sequencing showed a heterozygous missense variant c. 865A>G; p.Ile289Val in *CACNA1S* in patients 1–7. It was not found in the normal control and the unaffected family member (I-2; family 2) (Figure 7). This variant was previously reported in unrelated Thai patients in the original report [19]. The amino acid residue 289Ile is highly conserved in vertebrates [19]. According to gnomAD, the allele frequency is 0.00003185. It is not reported in South Asian and East Asian populations. This variant was not found in our in-house exome database of 1016 people with different phenotypes.

### 2.3. Immunohistochemistry of Cacna1s during Early Tooth Development

All animal experiments were reviewed and approved by the Niigata University Institutional Animal Care and Use Committee (approval number SA00610, SD01308). CD-1-strain mice were used in this study. Embryo heads were fixed in 4% buffered paraformaldehyde, wax embedded in paraffin, and serially sectioned at 7 µm. Paraffin sections were incubated with the antibodies against CACNA1S (ThermoFisher, Waltham, MA, USA; MA3-920, 1:100). The tyramide signal amplification system (Parkin Elmer Life Science, Waltham, MA, USA) with biotinylated horse antibodies (Vector, BA-2001, 1:100) was used for detecting the CACNA1S antibody. As a negative control, normal mouse serum was used instead of primary antibody.

#### Cacna1s Expression

**Figure 8:**
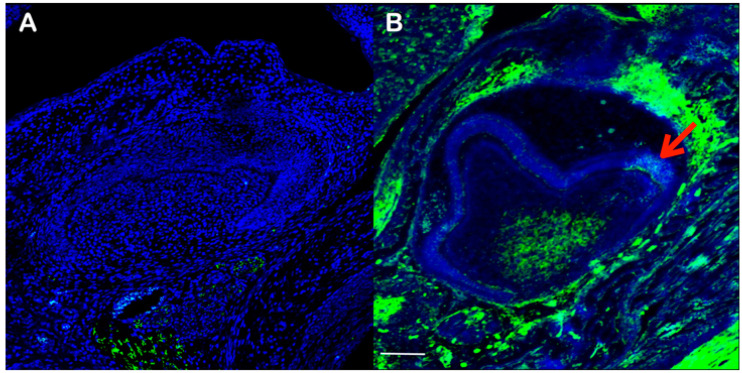
Frontal section showing immunohistochemistry of Cacna1S in wild-type mouse at embry- onic day 17.5 (**B**). Negative control (**A**). Arrow in B indicates Cacna1s expression at the secondary enamel knot. Scale bar: 100 μm.

Cacna1s expression was observed in the secondary enamel knot, which regulates cusp formation, while it could not be found in other parts of the tooth epithelium (Figure 8). Cacna1s was expressed in the caudal part of the dental papillae, but not in the cranial part. No expression of Cacna1s was observed in the stellate reticulum. Cacna1s was strongly expressed in mesenchyme at the collar region of the tooth germ.

## 3. Discussion

We identified a heterozygous missense variant c. 865A>G; p.Ile289Val in *CACNA1S* in seven affected patients, but not in the unaffected family member and control, suggestive of co-segregation of the genotype and phenotype. Dental anomalies caused by the heterozygous variant p.Ile289Val in *CACNA1S* appear to be inherited as an autosomal-dominant inheritance with complete penetrance. This variant has been reported once in Thai patients affected with similar dental anomalies [19]. *CACNA1S* encodes the pore-forming subunit of the dihydropyridine channel. The variant is located in the first pore-forming intramembrane domain between segment 5 and segment 6 of transmembrane domain 3, close to the three amino acid residues involved in calcium selectivity [19]. Therefore, the dental anomalies were likely the results of abnormal calcium selectivity and subsequent disruptive calcium homeostasis.

Tooth is formed by a sequence of epithelial–mesenchymal interactions of dental epithelium and neural crest-derived mesenchyme. Developing tooth germs go through a number of developmental stages prior to mineralization. The development of tooth shape requires the spatial and temporal control of the primary and secondary enamel knot signaling centers, the non-proliferative signaling centers [20]. Each tooth is considered to be highly self-regulated. The number and patterning of cusps are determined by the iterative activation of secondary enamel knots, which provide positional information via inductive interaction between the dental epithelium and the underlying mesenchymal cells [10,14]. Enamel knots regulate the growth and folding of the inner dental epithelium, which subsequently determines the final form and size of the tooth crown [10,14]. Differential growth and folding of the dental epithelium determine size and cusp patterns. The apoptotic mechanism also plays an additional role in sculpturing the final shape and size of cusps [21]. The cusp number is regulated by the sizes of the teeth and cusps [13]. The activation and inhibition of signaling from enamel knots result in differential growth and folding of the dental epithelium within the tooth germ, and subsequently determine the cusp patterns and dimensions [7]. The transition from the bud to the cap stage is important because most of the tooth morphology is already determined at this stage [8]. Once teeth are completely formed, each tooth anatomically has its own identity [22].

Having multiple supernumerary cusps in the molars in our patients implies that supernumerary secondary enamel knots were additionally formed as a result of the *CACNA1S* variant. High expression of Cacna1s at the secondary enamel knot in developing mouse molars in our study supports the role of *CACNA1S* in cusp formation (Figure 8). Since tooth cusps and separation of developing roots are a result of physiological folding of Hertwig epithelial root sheath, a dental epithelium [10,13]. Therefore, the dental malformations, including abnormal cusps, single-rooted molar, and taurodontism seen in our patients highlight the importance of proper calcium homeostasis in folding the dental epithelium.

The final shape of tooth crown depends on the number of secondary enamel knots and where they are located within the tooth germ. The shape of molars is determined by number, shape, and sizes of the cusps [10,13]. The round-shaped molars in our patients were likely the consequence of having so many supernumerary cusps because developmentally, cusps are formed first, and the formation process spreads centrifugally towards the apical end of the tooth. The supernumerary cusps in the molars in our patients appear smaller than the normal cusps. This was likely the consequence of having so many cusps on the limited occlusal surface area and the positions and proportions of the earlier developing cusps have great influence on the later developing ones within the same molar tooth [10,12]. In other words, cusp size dictates the cusp number, and the number of cusps is determined by tooth size [13]. The development of the single-cusped premolars in the patients might imitate that of ball python or bearded dragon, as their inner dental epithelium does not undergo folding; therefore, a tooth with a single prominent cusp is formed [23].

The *CACNAS* variant seems to cause impaired dental epithelial folding; too much folding in the molars and less folding in the premolars; and delayed folding (invagination) of Hertwig epithelial root sheath, which resulted in single-rooted molars or taurodontism. Apparently, the dental epithelium functions differently when it forms the crown or the root, depending on its intrinsic factors. Evidently, the consequences of the altered *CACNA1S* are different between incisors, premolars, and molars, suggestive of differential regulatory roles of *CACNA1S* in different teeth. Since calcium influx appears to have an influence on tooth morphogenesis, it is suggested that the study of the effects of calcium channel blockers taken during pregnancy on developing teeth should be performed.

Pathologies, such as the presence of multiple supernumerary cusps or reawakening of the potential to form more cusps, can be linked with Evo-Devo to elucidate a better understanding of how tooth structures, especially tooth cusps, form during development and through evolution [2].

## 4. Conclusions

In conclusion, the genetic variant (c. 865A>G; p.Ile289Val) in the *CACNA1S* gene is associated with abnormal crown and root morphology. Our observation suggests that crown morphology may be able to predict root morphology. Therefore, patients with atypical crown morphology should be referred for radiographs to assess root shape.

## Data Availability

Not applicable.
